# Latent Profile Analysis of Neuropsychiatric Symptoms and Cognitive Function of Adults 2 Weeks After Traumatic Brain Injury: Findings From the TRACK-TBI Study

**DOI:** 10.1001/jamanetworkopen.2021.3467

**Published:** 2021-03-30

**Authors:** Benjamin L. Brett, Mark D. Kramer, John Whyte, Michael A. McCrea, Murray B. Stein, Joseph T. Giacino, Mark Sherer, Amy J. Markowitz, Geoffrey T. Manley, Lindsay D. Nelson, and the TRACK-TBI Investigators, Opeolu Adeoye, Neeraj Badjatia, Kim Boase, Jason Barber, Yelena Bodien, M. Ross Bullock, Randall Chesnut, John D. Corrigan, Karen Crawford, Ramon Diaz-Arrastia, Sureyya Dikmen, Ann-Christine Duhaime, Richard Ellenbogen, V. Ramana Feeser, Adam R. Ferguson, Brandon Foreman, Raquel Gardner, Etienne Gaudette, Luis Gonzalez, Shankar Gopinath, Rao Gullapalli, J. Claude Hemphill, Gillian Hotz, Sonia Jain, C. Dirk Keene, Frederick K. Korley, Joel Kramer, Natalie Kreitzer, Harvey Levin, Chris Lindsell, Joan Machamer, Christopher Madden, Alastair Martin, Thomas McAllister, Randall Merchant, Pratik Mukherjee, Laura B. Ngwenya, Florence Noel, David Okonkwo, Eva Palacios, Ava Puccio, Miri Rabinowitz, Claudia Robertson, Jonathan Rosand, Angelle Sander, Gabriella Satris, David Schnyer, Seth Seabury, Sabrina Taylor, Nancy Temkin, Arthur Toga, Alex Valadka, Mary Vassar, Kevin Wang, John K. Yue, Esther Yuh, Ross Zafonte

**Affiliations:** 1 Department of Neurosurgery, Medical College of Wisconsin, Milwaukee; 2 Department of Neurology, Medical College of Wisconsin, Milwaukee; 3 Independent consultant; 4 Moss Rehabilitation Research Institute, Elkins Park, Pennsylvania; 5 Department of Psychiatry, University of California, San Diego, La Jolla; 6 Department of Family Medicine and Public Health, University of California, San Diego, La Jolla; 7 VA San Diego Healthcare System, San Diego, California; 8 Department of Physical Medicine and Rehabilitation, Harvard Medical School, Boston, Massachusetts; 9 Spaulding Rehabilitation Hospital, Charlestown, Massachusetts; 10 TIRR Memorial Hermann, Department of Physical Medicine and Rehabilitation, Baylor College of Medicine, Houston, Texas; 11 Department of Neurological Surgery, University of California, San Francisco, San Francisco; 12 University of Cincinnati, Cincinnati, Ohio; 13 University of Maryland, College Park; 14 University of Washington, Seattle; 15 Massachusetts General Hospital, Boston; 16 University of Miami, Florida; 17 Ohio State University, Columbus; 18 University of Southern California, Los Angeles; 19 University of Pennsylvania, Philadelphia; 20 MassGeneral Hospital for Children, Boston, Massachusetts; 21 Virginia Commonwealth University, Richmond; 22 University of California, San Francisco; 23 TIRR Memorial Hermann, Houston, Texas; 24 Baylor College of Medicine, Houston, Texas; 25 University of California, San Diego; 26 University of Michigan, Ann Arbor; 27 Vanderbilt University, Nashville, Tennessee; 28 UT Southwestern Medical Center, Dallas, Texas; 29 Indiana University, Bloomington; 30 University of Pittsburgh, Pittsburgh, Pennsylvania; 31 University of Texas at Austin, Austin, Texas; 32 University of Florida, Gainesville; 33 Harvard Medical School, Boston, Massachusetts

## Abstract

**Importance:**

Heterogeneity across patients with traumatic brain injury (TBI) presents challenges for clinical care and intervention design. Identifying distinct clinical phenotypes of TBI soon after injury may inform patient selection for precision medicine clinical trials.

**Objective:**

To investigate whether distinct neurobehavioral phenotypes can be identified 2 weeks after TBI and to characterize the degree to which early neurobehavioral phenotypes are associated with 6-month outcomes.

**Design, Setting, and Participants:**

This prospective cohort study included patients presenting to 18 US level 1 trauma centers within 24 hours of TBI from 2014 to 2019 as part of the Transforming Research and Clinical Knowledge in TBI (TRACK-TBI) study. Data were analyzed from January 28, 2020, to January 11, 2021.

**Exposures:**

TBI.

**Main Outcomes and Measures:**

Latent profiles (LPs) were derived from common dimensions of neurobehavioral functioning at 2 weeks after injury, assessed through National Institutes of Health TBI Common Data Elements (ie, Brief Symptom Inventory-18, Patient Health Questionnaire-9 Depression checklist, Posttraumatic Stress Disorder Checklist for DSM-5, PROMIS Pain Intensity scale, Insomnia Severity Index, Rey Auditory Verbal Learning Test, Wechsler Adult Intelligence Scale–Fourth Edition Coding and Symbol Search subtests, Trail Making Test, and NIH Toolbox Cognitive Battery Pattern Comparison Processing Speed, Dimensional Change Card Sort, Flanker Inhibitory Control and Attention, and Picture Sequence Memory subtests). Six-month outcomes were the Satisfaction With Life Scale (SWLS), Quality of Life after Brain Injury-Overall Scale (QOLIBRI-OS), Glasgow Outcome Scale-Extended (GOSE), and Rivermead Post-Concussion Symptoms Questionnaire (RPQ).

**Results:**

Among 1757 patients with TBI included, 1184 (67.4%) were men, and the mean (SD) age was 39.9 (17.0) years. LP analysis revealed 4 distinct neurobehavioral phenotypes at 2 weeks after injury: emotionally resilient (419 individuals [23.8%]), cognitively impaired (368 individuals [20.9%]), cognitively resilient (620 individuals [35.3%]), and neuropsychiatrically distressed (with cognitive weaknesses; 350 individuals [19.9%]). Adding LP group to models including demographic characteristics, medical history, Glasgow Coma Scale score, and other injury characteristics was associated with significantly improved estimation of association with 6-month outcome (GOSE *R^2^* increase = 0.09-0.19; SWLS *R^2^* increase = 0.12-0.22; QOLIBRI-OS *R^2^* increase = 0.14-0.32; RPQ *R^2^* = 0.13-0.34).

**Conclusions and Relevance:**

In this cohort study of patients with TBI presenting to US level-1 trauma centers, qualitatively distinct profiles of symptoms and cognitive functioning were identified at 2 weeks after TBI. These distinct phenotypes may help optimize clinical decision-making regarding prognosis, as well as selection and stratification for randomized clinical trials.

## Key Points

**Question:**

Are distinct phenotypes of neurobehavioral function identifiable 2 weeks after traumatic brain injury (TBI), and are phenotypes associated with 6-month quality of life and daily functioning?

**Findings:**

In this cohort study of 1757 patients with TBI, 4 distinct neurobehavioral phenotypes were identified 2 weeks after injury based on self-report and cognitive performance measures. Phenotypes were associated with improved estimations of 6-month outcomes compared with conventional medical record documentation and injury severity factors.

**Meaning:**

These findings suggest that a phenotype-based classification system could potentially be used as part of an applied precision medicine approach to reliably identify patients at risk for poor outcomes and in need of early, targeted intervention based on phenotypic characteristics following TBI.

## Teaser

This cohort study characterizes phenotypes of neuropsychiatric and cognitive functioning in patients 2 weeks after traumatic brain injury and assess whether these phenotypes are associated with 6-month outcomes.

## Introduction

Traumatic brain injury (TBI) results in approximately 2.8 million emergency department (ED) and hospital visits annually in the US and is associated with persistent consequences after discharge,^1^ including significant disability, caregiver burden, and socioeconomic impact.^2,3,4,5^ Approaches to classify the diverse TBI population at the time of injury have remained largely unchanged over the past several decades and have emphasized placing patients into 1 of, at most, 4 broad injury severity categories based on acute Glasgow Coma Scale (GCS) score and head computed tomography (CT) scans: uncomplicated mild (GCS score, 13-15, with CT scans negative for acute intracranial findings), complicated mild (GCS score, 13-15, with CT scans positive for acute intracranial findings), moderate (GCS score, 9-12), and severe (GCS score, 3-8).^6,7^ However, TBI severity is only weakly correlated with functional outcomes (eg, Glasgow Outcome Scale–Extended [GOSE] scores)^8^ and unreliably associated with patient-reported outcomes, such as persistent TBI-related symptoms or quality of life (QOL).^9,10,11,12,13,14,15,16^ Outcome estimation is only modestly improved when additionally considering other routinely available information, such as demographic characteristics and premorbid factors.^17,18,19^ Unreliable associations between early risk factor variables and later outcomes, along with varied outcomes within TBI severity strata, imply heterogeneity across patients with TBI that must be better understood to target clinical care and to establish evidence-based treatments.^20^

Considering how patients present in varied neurobehavioral domains that can be affected by TBI, such as psychological and cognitive functioning, may be productive in identifying distinct clinical phenotypes of TBI. Inadequate TBI classification is hypothesized to be a prime reason why numerous TBI clinical trials have failed.^21,22,23^ Recent efforts to identify distinct clinical phenotypes based on patient-reported symptoms alone have demonstrated potential challenges owing to the high level of comorbidity among the diverse physical, cognitive, and emotional symptoms reported by patients.^24,25^ Considering broader arrays of clinical variables, such as self-report and more objective measures, may be valuable for discerning distinct subtypes of TBI. Using cluster analysis of a comprehensive neuropsychological battery, Sherer et al^26^ found several distinct patterns of neuropsychological function in community dwelling patients who had experienced TBI 7 years previously. These findings support the prospect of identifying clinically relevant subgroups of TBI earlier in the recovery period, while demonstrating the interpretive value of person-centered analytic approaches vs more widely used variable-centered approaches, such as factor analysis and regression modeling.

Latent profile analysis (LPA) is another person-centered analytic approach well-suited to categorize this heterogeneity with more granularity, as it classifies individuals into unmeasured latent groups based on discernable patterns of endorsement and functioning, such as symptoms and cognitive performance.^27^ With a goal to identify clinically distinct subgroups of TBI, we performed LPA on diverse neurobehavioral dimensions assessed 2 weeks after TBI in the multicenter Transforming Research and Clinical Knowledge in TBI (TRACK-TBI) study sample. TRACK-TBI followed patients with TBI across the injury severity spectra and assessed them with comprehensive neurobehavioral assessments (ie, self-report inventories and cognitive performance measures recommended by the National Institutes of Health [NIH] as Common Data Elements). The aim of this study was 2-fold: to determine whether distinct patterns (ie, LPs) of symptoms and cognitive functioning at 2 weeks after TBI can be identified and to determine if 2-week neurobehavioral LP group membership was associated with improved estimates of associations with 6-month outcomes of self-reported QOL, TBI-related symptoms, and functional limitations beyond routinely assessed characteristics (ie, demographic characteristics, psychiatric history, TBI injury characteristics).

## Methods

This cohort study met institutional review board approval at the Medical College of Wisconsin and University of California, San Francisco. All participants provided written informed consent prior to any study activities. This report was completed in accordance with the Strengthening the Reporting of Observational Studies in Epidemiology (STROBE) reporting guideline for cohort studies.

### Participants and Procedure

The TRACK-TBI study enrolled patients presenting with TBI at 18 level-1 trauma centers in the US from 2014 to 2019. Inclusion criteria were head trauma with altered mental status (ie, unconsciousness, peritraumatic amnesia, or other signs of altered consciousness), having a head CT scan ordered by the treating ED physician, and enrollment within 24 hours of injury. Exclusion criteria included being in police or other law enforcement custody or pregnant, having nonsurvivable physical trauma, serious and persistent mental illness, or neurologic disease, or not speaking English; however, some sites recruited Spanish-speaking participants. Exclusions for this analysis were age younger than 17 years, being assigned an abbreviated assessment battery owing to degree of impairment, withdrawal from the study, death before the 2-week follow-up, or unknown loss to follow-up (eFigure 1 in the Supplement).

### Neurobehavioral Functioning at 2 Weeks

#### Self-reported Symptom Measures

Symptoms of psychological distress were assessed with the 18-item Brief Symptom Inventory.^28^ Posttraumatic stress disorder symptoms were assessed with the Posttraumatic Stress Disorder Checklist for DSM-5.^29^ Depression was assessed with the 9-item Patient Health Questionnaire-9.^30^ Sleep quality and disturbance were assessed with the Insomnia Severity Index.^31^ Pain intensity was assessed with the PROMIS Pain Intensity scale.^32^

#### Cognitive Performance Measures

Cognitive performance tests were administered at 2 weeks after injury. Cognitive performance measures included verbal and visual episodic memory (Rey Auditory Verbal Learning Test; NIH Toolbox Picture Sequence Memory),^33^ processing speed (Wechsler Adult Intelligence Scale–Fourth Edition Coding and Symbol Search subtests; NIH Toolbox Pattern Comparison Processing Speed),^34^ and executive functioning (Trail Making Test; NIH Toolbox Dimensional Change Card Sort, Flanker Inhibitory Control, and Attention subtests).^35,36^

### Outcomes at 6 Months

Outcomes collected at 6 months after injury included measures of QOL and daily functioning. Patients reported general life satisfaction with the Satisfaction With Life Scale (SWLS),^37^ TBI-relevant health-related QOL with the Quality of Life after Brain Injury-Overall Scale (QOLIBRI-OS),^38^ TBI-related symptoms with the Rivermead Post-Concussion Symptoms Questionnaire (RPQ), and daily functioning with the GOSE.^39^

### Statistical Analyses

Statistical analyses were performed in *Mplus* statistical software version 8.3 (Muthén & Muthén) and SPSS Statistics version 24 (IBM). Self-reported symptom measures were consolidated into 6 correlated factors based on our factor analytic work with these data^40^ which found the 57 items to reflect 6 distinct dimensions: depression, anxiety, fear, sleep, physical symptoms, and pain (eFigure 2 in the Supplement). These dimensions were elevated among patients with TBI compared with orthopedic controls, suggesting they are preferentially associated with TBI. For the purpose of this study, factor scores on these dimensions were extracted in *Mplus*. Consistent with symptom reporting instruments,^40^ factor analysis was used to reduce the 15 cognitive performance indices into 5 dimensions (eFigure 3 and eAppendix 1 in the Supplement).

LPA is a Gaussian finite mixture modeling method used to identify distinct clusters (ie, profiles) based on participants’ responses to a set of measures or variables using maximum likelihood estimation.^27^ The LPA method assumes that participants’ membership within the underlying LP groups influence their responses on these measures. Determining the optimal profile model (ie, number of unique profiles) was based on simultaneous consideration of the bayesian information criterion^41^ index and modified Lo-Mendell-Rubin likelihood ratio tests,^42^ which have been recommended as the most effective methods in deciding on the number of latent classes or profile solutions based on Monte Carlo simulation (eAppendix 2 in the Supplement).^43^ Probability of LP membership was estimated based on responses to indicators submitted to LPA as described elsewhere,^27^ and participants were assigned to the LP with the highest posterior probability.

Associations between demographic characteristics (ie, age, self-reported race, sex, education), injury-related characteristics (eg, TBI Severity Group, LOC), and medical history factors (ie, mental health disorders) previously shown to be associated with outcomes after TBI (eg, cognitive, psychological) were also examined. Analysis of variance tests examined continuous variable differences across LP membership, with significant omnibus tests further interrogated through post-hoc Tukey honest significant difference test (Tukey HSD). Categorical variables were compared across LP groups with χ^2^ or Fisher exact tests. All analyses were performed in August 2020.

For each of the 4 outcomes assessed at 6 months (eAppendix 3 in the Supplement), 2 separate sets of linear regression models were fit. The first set of linear regression models (model 1) assessed the associations between common variables that can be found in medical records (demographic characteristics, psychiatric history, TBI characteristics, conventional TBI severity taxonomy) and each 6-month outcome. A second set of models (model 2) was then fit that included all of the variables from the first model, with LP membership (ie, group based on 2-week neurobehavioral profile) as an additional variable. This was performed to assess whether LP membership was a significant independent variable of 6-month outcomes in the context of other established prognostic factors and to examine the degree in which LP group membership accounts for additional variance in 6-month outcomes (ie, *R*^2^ value). To control for the influence of potential bias in missingness or loss to follow-up at 6-month visits, propensity weights were included within the regression models (eAppendix 4 in the Supplement). Curve estimation showed nonlinear (ie, quadratic) associations between age with each of the 6-month outcomes, as well as for education and SWLS. As such, respective polynomial terms based on the aforementioned associations were included within regression models. *P* values were 2-sided. Multiple comparison correction was applied to the 8 overall models (4 iterations of models 1 and 2) using a Bonferroni adjustment, resulting in statistical significance being evaluated at the *P* = .006 level (0.05 / 8 = .006).

## Results

### Participant Characteristics

Among 2698 patients presenting at participating trauma centers, 1757 patients (mean [SD] age, 39.9 [17.0] years, 1184 [67.4%] men) met inclusion criteria and had sufficient 2-week neurobehavioral data for inclusion (eFigure 1 in the Supplement). Table 1 displays demographic, medical history, and injury characteristics for the sample evaluated at 2 weeks. Patients included 1351 (76.9%) White individuals, with a mean (SD) of 14.3 (7.9) years of education. Using traditional categories for classifying TBI severity based on admission Glasgow Coma Scale (GCS) score and acute intracranial findings on clinical head CT scans, the sample included 1021 patients (61.4%) with GCS 13 to 15 without acute intracranial CT findings, 511 patients (30.7%) with GCS 13 to 15 and with acute intracranial CT findings, 50 patients (3.0%) with GCS 9 to 12, and 80 (4.8%) GCS with 3 to 8.

**Table 1.** Demographic, History, and Injury Characteristics of Patients Presenting With TBI

Characteristic	No. (%)					*F* or χ^2^ statistic^a^	*P* value
	All TBI (n = 1757)	Latent profile					
		Emotionally resilient (n = 419)	Cognitively impaired (n = 368)	Cognitively resilient (n = 620)	Neuropsychiatrically distressed (n = 350)		
Age, mean (SD), y	39.9 (17.0)	41.7 (18.4)	48.3 (18.1)	35.2 (14.5)	37.5 (14.0)	52.3	<.001
Sex							
Men	1184 (67.4)	316 (75.4)	274 (74.5)	398 (64.2)	196 (56.0)	42.6	<.001
Women	573 (32.6)	103 (24.6)	94 (25.5)	222 (35.8)	154 (44.0)		
Race							
White	1351 (76.9)	337 (80.4)	280 (76.1)	494 (79.7)	240 (68.6)	62.4	<.001
Black	291 (16.6)	47 (11.2)	72 (19.6)	74 (11.9)	98 (28.0)		
Other or unknown^b^	115 (6.5)	35 (8.4)	16 (4.3)	52 (8.4)	12 (3.4)		
Education, mean (SD), y	14.3 (7.9)	14.8 (7.8)	13.7 (9.4)	14.9 (7.7)	13.0 (6.3)	10.1	<.001
Psychiatric history	393 (22.4)	44 (10.5)	73 (19.9)	147 (23.7)	129 (36.9)	76.6	<.001
Cause of injury							
MVC	1012 (57.6)	202 (48.2)	190 (51.6)	405 (65.3)	215 (61.4)		
Fall	459 (26.1)	145 (34.6)	117 (31.8)	129 (20.8)	68 (19.4)	69.92	<.001
Assault or violence	120 (6.9)	17 (4.1)	27 (7.3)	36 (5.8)	40 (11.4)		
Other or missing	166 (9.4)	55 (13.1)	34 (9.2)	50 (8.1)	27 (7.8)		
Highest level of care							
Emergency department	458 (26.1)	111 (26.5)	66 (17.9)	192 (31.0)	89 (25.4)	53.4	<.001
Inpatient ward	719 (40.9)	171 (40.8)	127 (34.5)	261 (42.1)	160 (45.7)		
ICU	580 (33.0)	137 (32.7)	175 (47.6)	167 (26.9)	101 (28.9)		
TBI severity group							
GCS 13-15						65.2	<.001
CT−	1021 (61.4)	230 (58.2)	149 (44.5)	402 (67.2)	240 (68.6)		
CT+	511 (30.7)	134 (33.9)	135 (40.3)	167 (27.9)	75 (21.4)		
GCS 9-12	50 (3.0)	11 (2.8)	21 (6.3)	9 (1.5)	9 (2.6)		
GCS 3-8	80 (4.8)	20 (5.1)	30 (9.0)	20 (3.3)	10 (2.9)		
LOC^c^	1460 (83.1)	356 (85.2)	295 (80.2)	506 (82.0)	303 (86.5)	19.3	<.01
PTA^c^	1292 (73.6)	299 (71.3)	270 (73.3)	457 (73.7)	266 (76.0)	10.31	.11

Abbreviations: CT, computed tomography present (+) or absent (−) for acute intracranial findings within 24 hours after injury; GCS, Glasgow Coma Scale score (at admission); ICU, intensive care unit; LOC, loss of consciousness; MVC, motor vehicle collision; PTA, posttraumatic amnesia; TBI, traumatic brain injury.

^a^ LP group differences across demographic, health history, and injury characteristics were testing using analysis of variance (*F*-statistic provided for continuous variables) and χ^2^ tests for categorical variables.

^b^ Includes Asian, native Hawaiian or other Pacific Islander, American Indian or Alaska Native, or individuals who did not report their race.

^c^ Witnessed and suspected LOC and PTA categories are collapsed.

### Cognitive Factors

A 5-factor solution of the cognitive performance variables was deemed to best balance considerations of model fit and parsimony. These correlated factors reflected speed, executive function, early memory, intermediate memory, and delayed memory. eFigure 3 and eAppendix 5 in the Supplement provide additional details about the configuration and fit of the model. Factor scores for the 5-factor CFA model were extracted on the full sample for use in LPA.

### LPA

**Figure 1.**
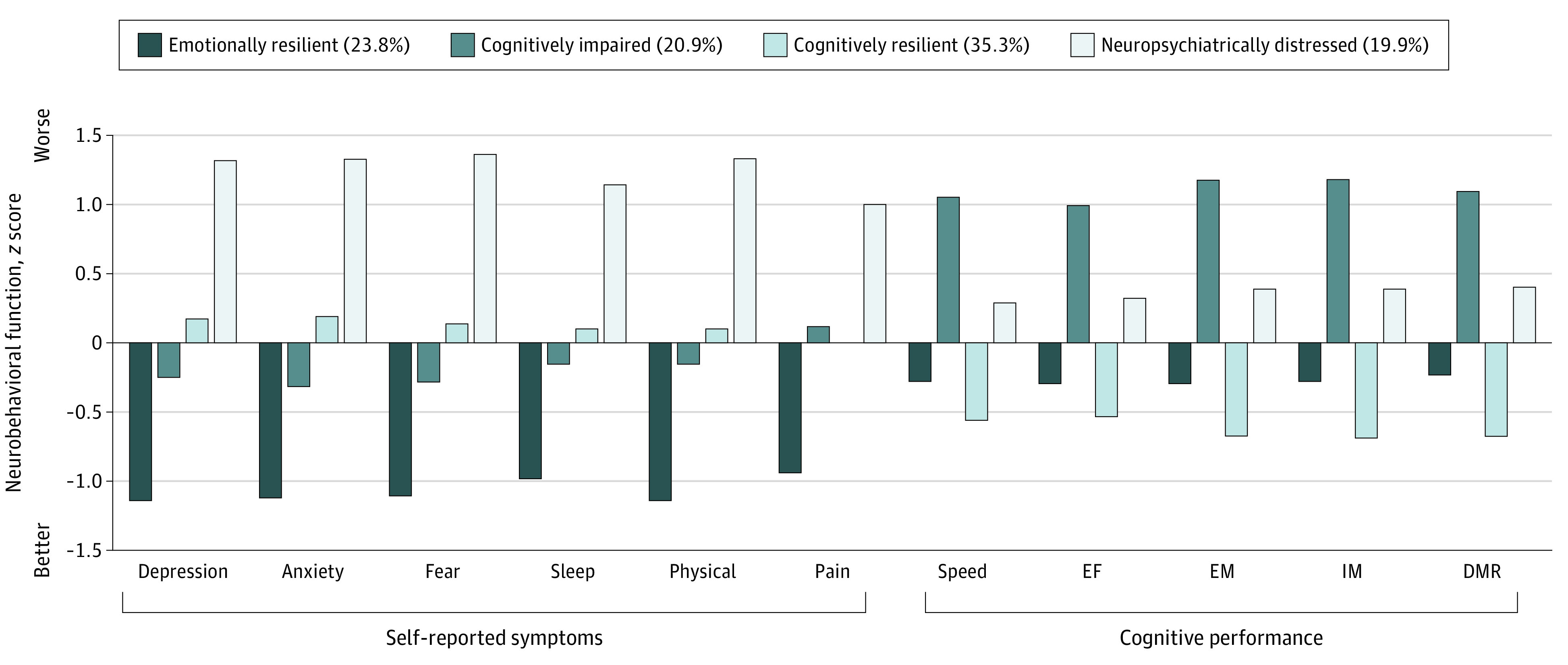
Mean Standardized Factor Score on Each Neurobehavioral Dimension at 2 Weeks After Injury, Stratified by Latent Profile Group Higher scores indicate worse functioning (ie, more symptoms, poorer cognitive functioning); DMR, delayed memory recall; EF, executive function; EM, early memory; IM, intermediate memory.

LPA showed that a single profile (ie, treating all participants as part of 1 homogeneous group based on 2-week neurobehavioral characteristics) provided substantially poorer fit than solutions with multiple LPs (eTable in the Supplement). A 4-LP solution was selected as the optimal model to balance the ability to discern groups and model parsimony (eAppendix 6 in the Supplement). Based on the mean scores across neurobehavioral dimensions for each profile (Figure 1), LP groups were labeled emotionally resilient (419 patients [23.8%]): cognitively impaired (368 patients [20.9%]), cognitively resilient (620 patients [35.3%]), and neuropsychiatrically distressed (with cognitive weaknesses; 350 patients [19.9%]).

### Comparisons of LPs on Demographic, Medical History, and Injury Characteristics

Significant differences in age, education, sex, race, and prior psychiatric diagnosis were observed across LPs (Table 1). The most notable differences involved age (cognitively impaired: mean [SD] age, 48.3 [18.1] years vs cognitively resilient: mean [SD] age, 35.2 [14.5] years), sex (emotionally resilient: 316 [75.4%] men vs neuropsychiatrically distressed: 196 [56.0%] men), and history of mental health disorder (emotionally resilient: 44 patients [10.5%] vs neuropsychiatrically distressed: 129 patients [36.9%]). For injury-related characteristics, the LPs significantly differed in most markers of TBI or injury severity (ie, GCS scores, TBI Severity Group, highest level of care, and loss of consciousness). Generally, the cognitively impaired group exhibited greater injury severity, such as higher rates of ICU admission (175 patients [47.6%] vs 137 patients [32.7%] in the emotionally resilient group, 167 patients [26.9%] in the cognitively resilient group, and 101 patients [28.9%] in the neuropsychiatrically distressed group; *P* < .001) and lower admission GCS scores (51 patients [15.3%] with GCS score <13 vs 31 patients [7.9%] in the emotionally resilient group, 29 patients [4.8%] in the cognitively resilient group, and 19 patients [5.5%] in the neuropsychiatrically distressed group; *P* < .001).

### Incremental Value of Neurobehavioral Profiles in 6-Month Outcomes

**Figure 2.**
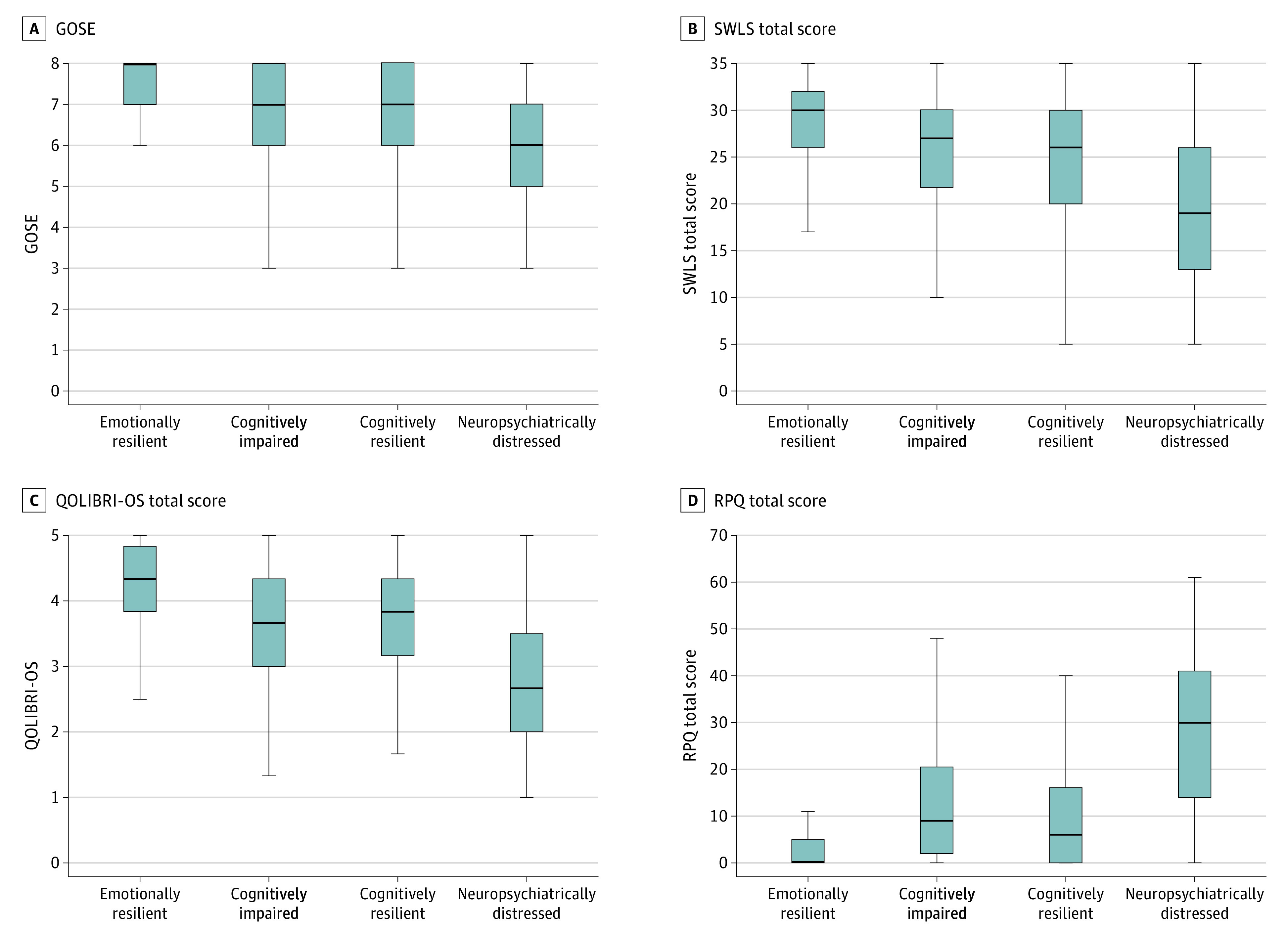
Six-Month Outcomes Stratified by 2-Week Neurobehavioral Latent Profile Group Lines indicate median; boxes, interquartile range; error bars, range; GOSE, Glasgow Outcome Scale-Extended; QOLIBRI-OS, Quality of Life after Brain Injury-Overall Scale; RPQ, Rivermead Post-Concussion Symptoms Questionnaire; and SWLS, Satisfaction With Life Scale.

Across all 6-month outcome measures, linear regression model 1 showed significant but weak associations (*R*^2^ = 0.08-0.13) from the combination of widely used independent variables of age, education, sex, race, psychiatric history, and TBI severity group (Table 2 and Table 3). TBI severity (according to GCS grouping) was not significantly associated with 6-month ratings of TBI-related QOL (QOLIBRI-OS score), SWLS, or self-reported TBI-related symptoms (RPQ). Adding LP group to these models (model 2) demonstrated significant incremental utility of considering neurobehavioral profiles for all outcomes (Figure 2). Adding LP group increased the amount of variance explained for all outcomes approximately 2-fold or more (increase in SWLS *R^2^* = 0.12-0.22; QOLIBRI-OS *R^2^* = 0.14-0.32; RPQ *R^2^* = 0.13-0.34; GOSE *R^2^* = 0.090.19).

**Table 2.** Latent Profiles and 6-Month Outcomes of Well-being

Outcome	SWLS				QOLIBRI-OS			
	Model 1, B (95% CI)^a^	*P* value	Model 2, B (95% CI)^b^	*P* value	Model 1, B (95% CI)^a^	*P* value	Model 2, B (95% CI)^b^	*P* value
*F*	18.41	<.001	26.40	<.001	21.37	<.001	47.16	<.001
*R*^2^	0.12		0.22		0.14		0.32	
Age (y)	−0.02 (−0.04 to .01)	.27	−0.031 (−0.056 to −0.001)	.03	−0.009 (−0.012 to −0.005)	<.001	−0.009 (−0.013 to −0.003)	.001
Age (quadratic)	0.02 (<0.01 to 0.01)	.03	0.001 (−0.001 to 0.002)	.42	<0.001 (<0.001 to 0.001)	<.001	<0.001 (<−0.001 to<−0.001)	.01
Education (y)	0.36 (0.23 to 0.50)	<.001	0.22 (0.09 to 0.36)	.001	0.06 (0.04 to 0.07)	<.001	0.022 (0.005 to 0.038)	.03
Education (quadratic)	0.04 (0.02 to 0.06)	.02	0.04 (0.02 to 0.06)	.001	NA		NA	
Sex	−0.09 (−0.91 to 0.73)	.83	0.46 (−0.32 to 1.24)	.25	−0.13 (−0.21 to −0.03)	.01	−0.11 (−0.23 to 0.01)	.06
Race (vs White)								
Unknown	0.15 (−1.39 to 1.69)	.85	0.11 (−1.35 to 1.56)	.89	0.04 (−0.16 to 0.24)	.68	0.23 (.01 to 0.44)	.04
Black	−3.47 (−4.52 to −2.41)	<.001	−2.70 (−3.71 to −1.69)	<.001	−0.37 (−0.51 to −0.23)	<.001	−0.05 (−0.20 to 0.11)	.57
Psychiatric history	4.43 (3.50 to 5.36)	<.001	3.09 (2.19 to 3.99)	<.001	0.65 (0.53 to 0.77)	<.001	0.21 (0.07 to 0.34)	.003
TBI severity^c^								
GCS 13-15								
CT−	1.62 (−0.12 to 3.35)	.07	2.49 (0.83 to 4.16)	.003	0.01 (−0.22 to 0.24)	.91	0.59 (0.34 to 0.84)	<.001
CT+	1.78 (−0.04 to 3.60)	.06	2.42 (0.69 to 4.16)	.005	−0.05 (−0.28 to −0.20)	.72	0.42 (0.16 to 0.69)	.002
GCS 9-12	0.83 (−1.95 to 3.62)	.56	1.13 (−1.50 to 3.76)	.40	−0.04 (−0.41 to 0.33)	.83	0.13 (−0.28 to 0.53)	.54
Latent profile ^d^								
Emotionally resilient	NA	NA	7.46 (6.28 to 8.63)	<.001	NA	NA	1.07 (0.89 to 1.25)	<.001
Cognitively impaired	NA	NA	5.12 (3.93 to 6.32)	<.001	NA	NA	0.40 (0.21 to 0.59)	<.001
Cognitively resilient	NA	NA	3.62 (2.57 to 4.67)	<.001	NA	NA	0.63 (0.47 to 0.79)	<.001

Abbreviations: CT, computed tomography; GCS, Glasgow Coma Scale; NA, not applicable; QOLIBRI-OS, Quality of Life after Brain Injury-Overall Scale; SWLS, Satisfaction With Life Scale; TBI, traumatic brain injury.

^a^ Model 1 independent variables were age, sex, race, education, psychiatric history (reference group is those with a psychiatric history), and TBI severity.

^b^ Model 2 entered the same independent variables as model 1 with the addition of neurobehavioral latent class membership.

^c^ Reference Group is severe TBI (GCS 3-8).

^d^ Reference group is neuropsychiatrically distressed class.

**Table 3.** Latent Profiles and 6-Month Outcomes of Function and TBI Symptoms

Outcome	GOSE				RPQ			
	Model 1, B (95% CI)^a^	*P* value	Model 2, B (95% CI)^b^	*P* value	Model 1, B (95% CI)^a^	*P* value	Model 2, B (95% CI)^b^	*P* value
*F*	11.80	<.001	21.44	<.001	20.34	<.001	51.19	<.001
*R*^2^	0.09		0.19		0.13		0.34	
Age (y)	−0.007 (−0.011 to −.003)	<.001	−0.007 (−0.011 to −0.003)	.001	0.01 (0.01 to 0.15)	<.001	0.10 (0.01 to 0.15)	<.001
Age (quadratic)	<0.001 (<0.001 to 0.001)	<.001	<0.001 (<−0.001 to <0.001)	.01	−0.007 (−0.009 to 0.004)	<.001	−0.004 (−0.006 to −0.002)	<.001
Education (y)	0.06 (0.04 to 0.08)	<.001	0.024 (0.003 to 0.045)	.003	−0.86 (−1.11 to −0.60)	<.001	−0.30 (−0.54 to −0.07)	.01
Sex	−0.16 (−0.29 to −0.04)	.01	−0.11 (−0.23 to 0.01)	.06	3.57 (2.02 to 5.12)	<.001	2.37 (1.00 to 3.74)	.001
Race (vs White)								
Not reported	0.22 (0.01 to 0.44)	.06	0.23 (0.01 to 0.44)	.04	−1.39 (−4.31 to 1.52)	.35	−1.16 (−3.72 to 1.39)	.37
Black or other	−0.21 (−0.37 to −0.05)	.009	−0.05 (−0.20 to 0.11)	.57	5.90 (3.91 to 7.89)	<.001	3.14 (1.37 to 4.91)	.001
Psychiatric history^a^	0.39 (0.25 to 0.53)	<.001	0.21 (0.07 to 0.34)	.003	−7.22 (−8.98 to −5.46)	<.001	−3.48 (−5.07 to −1.90)	<.001
TBI severity^c^								
GCS 13-15								
CT−	0.56 (0.30 to 0.83)	<.001	0.59 (0.34 to 0.84)	<.001	0.03 (−3.28 to 3.34)	.99	−1.29 (−4.23 to 1.64)	.39
CT+	0.40 (0.13 to 0.68)	.004	0.42 (0.16 to 0.69)	.002	0.80 (−2.67 to 4.27)	.65	0.01 (−3.06 to 3.07)	.99
GCS 9-12	0.04 (−0.38 to 0.47)	.85	0.13 (−0.28 to 0.53)	.54	0.50 (−4.81 to 5.81)	.85	−0.70 (−5.34 to 3.96)	.77
Laten profile membership^d^								
Emotionally resilient	NA	NA	1.07 (0.89 to 1.25)	<.001	NA	NA	−20.77 (−22.81 to −18.68)	<.001
Cognitively impaired	NA	NA	0.40 (0.21 to 0.59)	<.001	NA	NA	−12.71 (−14.82 to −10.60)	<.001
Cognitively resilient	NA	NA	0.63 (0.47 to 0.79)	<.001	NA	NA	−14.13 (−15.98 to −12.29)	<.001

Abbreviations: CT, computed tomography; GCS, Glasgow Coma Scale; GOSE, Glasgow Outcome Scale-Extended; NA, not applicable; RPQ, Rivermead Post-Concussion Symptoms Questionnaire; TBI, traumatic brain injury.

^a^ Model 1 independent variables were age, sex, race, education, psychiatric history (reference group is those with a psychiatric history), and TBI severity.

^b^ Model 2 entered the same independent variables as model 1 with the addition of neurobehavioral latent profile membership.

^c^ Reference Group is severe TBI (GCS 3-8).

^d^ Reference group is neuropsychiatrically distressed class.

## Discussion

This prospective, multicenter cohort study identified distinct subgroups of patients at 2 weeks post-TBI, characterized by qualitatively different patterns of neuropsychiatric symptoms and cognitive functioning. While these distinct neurobehavioral subgroups overlapped to some degree with traditional TBI severity groups, they exhibited greater associations with multiple 6-month outcomes, including TBI-related symptoms, QOL, and day-to-day functioning and independence. This early identification of distinct TBI phenotypes may help improve patient classification and potentially project outcome trajectories, which are critical to clinical trial design and achieving a precision medicine approach to TBI treatment. As approximately 40% of patients with mild TBI with acute intercranial findings on CT scans do not receive follow-up care with a practitioner^44^ and more than 50% report persistent life difficulties at 6 months after TBI,^45^ the need for triage to referral and treatment is clear, and would be of significant clinical and public health benefit.

Essentially all national health agencies (eg, NIH, National Cancer Institute) have placed a strong emphasis on advancing a precision medicine approach to clinical care and research studies.^40,46,47^ The potential for social and behavioral sciences to meaningfully contribute to the precision medicine initiative has been highlighted as well.^47,48^ The novel patient classification system based on comprehensive neurobehavioral measures (ie, latent profiles) identified in this study is a fundamental demonstration of the utility of social and behavioral sciences in advancing precision medicine. Specifically, we identified a patient classification system that involves 4 distinct phenotypes at 2 weeks after TBI using a comprehensive, multidimensional clinical outcome assessment battery. This novel classification system provides the potential to more accurately stratify patients for clinical trial enrollment subacutely after TBI, complement genetic and biological (eg, serum biomarkers, advanced magnetic resonance imaging) endophenotypes, support identification of personalized interventions, and aid in outcome prognosis based on expected trajectories.

Previous critiques, such as a 2017 analysis by Nelson et al,^49^ have suggested that conventional patient classification (based on GCS and CT indicators) after TBI may be too crude in patient stratification of clinical trials or outcome-based research, given that a diverse range of outcomes can occur within and across these classification groups. For example, individuals with mild TBI (GCS score, 13-15) have been observed as reporting greater rates of insomnia 1 year after TBI and beyond, compared with moderate and severe injuries.^11,50,51^ The distinct phenotypes identified in this study may improve prognostic heterogeneity beyond these conventional methods through more accurate patient classification in the subacute period after TBI, while still offering the parsimony and clinical utility that a grouping-based classification approach provides. As a concrete example, the utility and strength of association with QOL on 1 measure of TBI-related QOL at 6 months after TBI was limited, as significant differences across the 4 groups were not observed. Conversely, the 4-phenotype LP classification based on comprehensive neuropsychological assessment was significantly associated with QOL ratings at 6 months after TBI. Importantly, the 4-phenotype classification method accounted for significant variability in all 6-month outcomes (ie, accounted for ≥2-fold the amount of variance as all other variables in the model). This exceeded explained variance in conventional classification methods, as well as demographic characteristics, history (ie, mental health diagnosis and education), and conventional TBI severity taxonomy.

As part of the precision medicine approach, optimizing targeted intervention based on patient-specific factors can be potentially enhanced through the 4-phenotype taxonomy by helping to identify which patient is most likely to benefit from which treatment. In other words, development of novel therapeutics may involve testing medications that target specific phenotypes to improve outcomes of functioning. As an example, medications that augment acetylcholinergic functioning in the subacute period have been observed to have long-term beneficial effects on declarative memory, and to a lesser degree, other domains of cognitive functioning (eg, processing speed, attention).^52,53^ However, meaningful medication effects (ie, ≥1-SD improvement) on cognitive performance were observed for less than half of a study sample administered such an agent in an RCT,^53^ with only small differences between the drug and placebo groups.^54^ Our findings suggest that a minority of TBI patients (21%) have a neurobehavioral profile characterized predominantly by cognitive impairments. Informed by this 4-phenotype classification, a similar clinical trial could be conducted enrolling only those most likely to benefit from the targeted therapy (ie, cognitive impairment subgroup), in turn, increasing the chances of success of the trial.

This study also contains implications for clinical practice in the assessment and treatment of patients after TBI. Beginning as early as 2 weeks, using comprehensive neuropsychological assessment, patients who would be classified in the cognitive impairment and psychiatric distress groups could be identified, and person-centered treatment plans could be formulated and initiated. This could include early cognitive rehabilitation, psychotropic medications, and nonpharmacological treatment (psychotherapy or behavioral modification).^55,56,57^ Given that membership in either of these 2 groups was associated with poorer QOL, TBI-related symptoms, and functional outcomes at 6 months, identification of and early intervention for individuals within these groups may help to improve long-term outcomes after TBI. Additionally, classification of patients into 1 of the 4 phenotypic groups could help with optimal allocation of clinic resources and identifying those most in need of follow-up (ie, less emphasis on the 2 resilient groups).

### Limitations

This study has some limitations. Enrollment occurred at level-1 trauma centers, and our results may not generalize to individuals who do not pursue care at such a center. The 4-phenotype classification was developed with a comprehensive neuropsychological battery administered at 2 weeks after TBI and may not generalize to data collected at other postinjury time points. While patients’ neurobehavioral profiles may be discernable with a more abbreviated battery, additional work would be needed to identify a sensitive but time-efficient assessment approach to classify patients’ early neurobehavioral profiles. This is particularly important in the context of randomized clinical trials, as past trials have often involved neuroprotective agents, which require administration within hours of injury and hospitalization. Future work should examine how medical history, biological, or injury-related variables may allow for preliminary classification of patients into these profiles at hospital admission. The longitudinal stability of the 4-phenotype classification taxonomy across the course of TBI recovery is also a direction for future studies. Unavoidably, our focus on neuropsychological performance restricted the sample to patients capable of participating in such assessment and therefore dismissed a substantial percentage of patients with severe TBI who could not engage in such testing at 2 weeks after injury. Ongoing work by the TRACK-TBI investigators will provide methods to minimize missing data owing to severity of cognitive impairment.

## Conclusions

In this large prospective cohort study of patients with recent TBI presenting at level 1 trauma center, 4 distinct clinical phenotypes of patients were identified based on various dimensions of neuropsychiatric symptoms and cognitive functioning at 2 weeks after injury. These distinct phenotypes were superior to conventional medical record documentation injury severity factors in their associations with QOL, TBI-related symptoms, and daily functioning 6 months after TBI. This and other possible methods of patient classification offer the potential to optimize patient selection, stratification, and prognostic appraisal as part of clinical trial and precision medicine practices.

## Notes

Supplement. **eFigure 1.** Recruitment and Retention Flow Chart **eFigure 2.** Factor Structure of Self-report Neuropsychiatric Symptoms **eFigure 3.** Factor Model of Cognitive Performance Measures **eAppendix 1.** Factor Analysis of Cognitive Measures **eAppendix 2.** Criteria for Determining Final Latent Profile Analysis Model **eAppendix 3.** Range and Interpretation of Distal Outcomes at 6 Months After TBI **eAppendix 4.** Propensity Weighting Procedure and Related Variables **eAppendix 5.** Factor Analysis Results of Cognitive Measures **eAppendix 6.** Results of the Latent Profile Analyses Model Fit **eTable.** Overall and Relative Fit of Models With Increasing Number of Latent Profiles **eReferences**
