# Supplementary material for: Latent Profile Analysis of Neuropsychiatric Symptoms and Cognitive Function of Adults 2 Weeks After Traumatic Brain Injury: Findings From the TRACK-TBI Study
Source: JAMA Netw Open. 2021 Mar 30;4(3):e213467. doi: 10.1001/jamanetworkopen.2021.3467 (PMC8010589; doi:10.1001/jamanetworkopen.2021.3467)
Supplement: Supplement. — eFigure 1. Recruitment and Retention Flow Chart eFigure 2. Factor Structure of Self-report Neuropsychiatric Symptoms eFigure 3. Factor Model of Cognitive Performance Measures eAppendix 1. Factor Analysis of Cognitive Measures eAppendix 2. Criteria for Determining Final Latent Profile Analysis Model eAppendix 3. Range and Interpretation of Distal Outcomes at 6 Months After TBI eAppendix 4. Propensity Weighting Procedure and Related Variables eAppendix 5. Factor Analysis Results of Cognitive Measures eAppendix 6. Results of the Latent Profile Analyses Model Fit eTable. Overall and Relative Fit of Models With Increasing Number of Latent Profiles eReferences [file jamanetwopen-e213467-s001.pdf]

Supplemental Online Content

Brett BL, Kramer MD, Whyte J, et al; TRACK-TBI Investigators. Latent profile analysis of neuropsychiatric symptoms and cognitive function of adults 2 weeks after traumatic brain injury: findings from the TRACK-TBI study. *JAMA Netw Open*. 2021;4(3):e213467. doi:10.1001/jamanetworkopen.2021.3467

eFigure 1. Recruitment and Retention Flow Chart
eFigure 2. Factor Structure of Self-report Neuropsychiatric Symptoms
eFigure 3. Factor Model of Cognitive Performance Measures
eAppendix 1. Factor Analysis of Cognitive Measures
eAppendix 2. Criteria for Determining Final Latent Profile Analysis Model
eAppendix 3. Range and Interpretation of Distal Outcomes at 6 Months After TBI
eAppendix 4. Propensity Weighting Procedure and Related Variables
eAppendix 5. Factor Analysis Results of Cognitive Measures
eAppendix 6. Results of the Latent Profile Analyses Model Fit
eTable. Overall and Relative Fit of Models With Increasing Number of Latent Profiles
eReferences

This supplemental material has been provided by the authors to give readers additional information about their work.

© 2021 Brett BL et al. *JAMA Network Open.*

eFigure 1. Recruitment and Retention Flow Chart

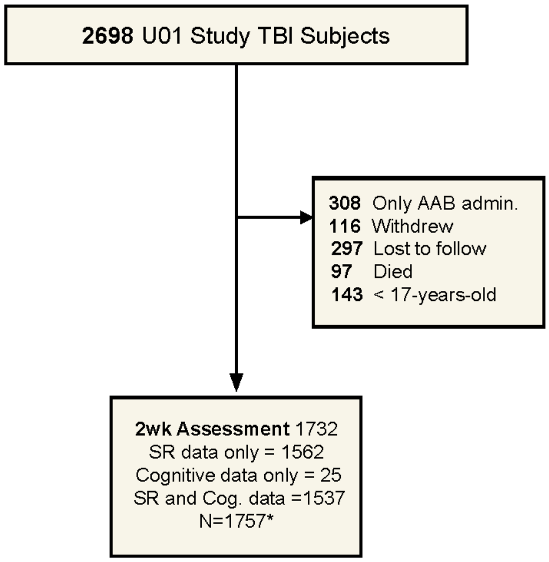

"SR" = Self-Report Measures

"AAB" = Abbreviated Assessment Battery (SR measures not administered due to patient level of impairment)

*A total of 1757 unique assessments contributed to estimation in latent profile analyses and related analyses

© 2021 Brett BL et al. *JAMA Network Open.*

eFigure 2. Factor Structure of Self-report Neuropsychiatric Symptoms

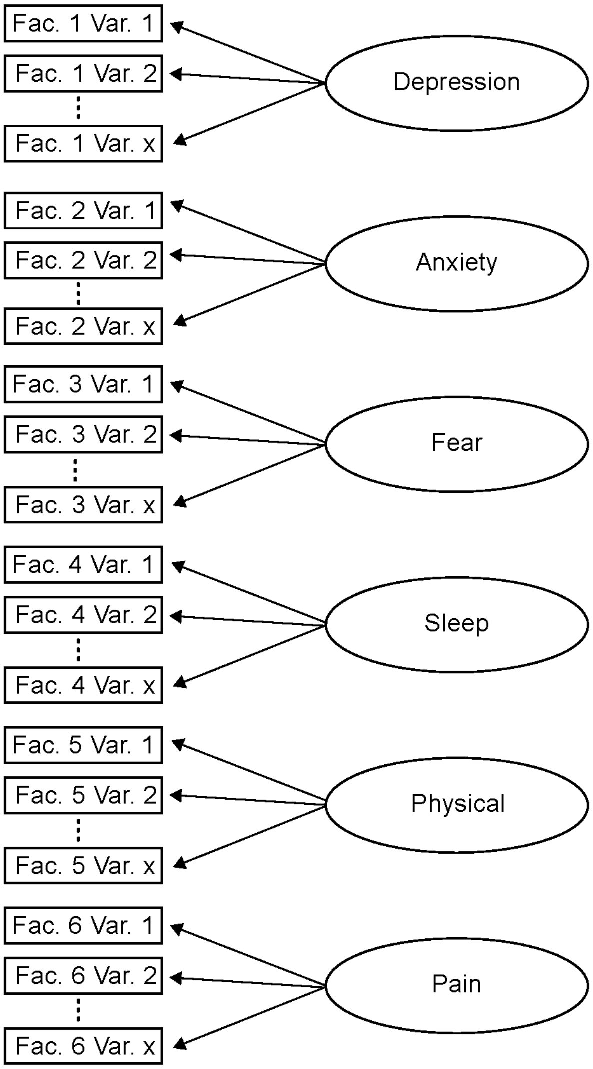

Factor structure of self-report neuropsychiatric symptoms. Factor scores were extracted from this previously established model for the present study's latent profile analysis.[1] The 57 items contributing to the model reflected those of the following National Institutes of Health TBI Common Data Elements: 18-item Brief Symptom Inventory (BSI-18), Post-Traumatic Stress Disorder Checklist for DSM-5 (PCL-5; 20 items), 9-item Patient Health Questionnaire depression checklist (PHQ-9), Insomnia Severity Index (ISI; 7 items), and PROMIS Pain Intensity Scale (3 items). As described in Nelson et al.,[1] the Depression factor comprised 14 items from the PHQ-9, PCL-5, and BSI-18; Anxiety comprised 7 PCL-5 items; Fear comprised 13 items from the PCL-5 and BSI-18; Sleep comprised 9 items from the ISI, PHQ-9, and PCL-5; Physical comprised 10 items form the PHQ-9, PCL-5, and BSI-18; and Pain comprised 4 items from the BSI-18 (chest pain) and PROMIS Pain Intensity Scale.

© 2021 Brett BL et al. *JAMA Network Open.*

eFigure 3. Factor Model of Cognitive Performance Measures

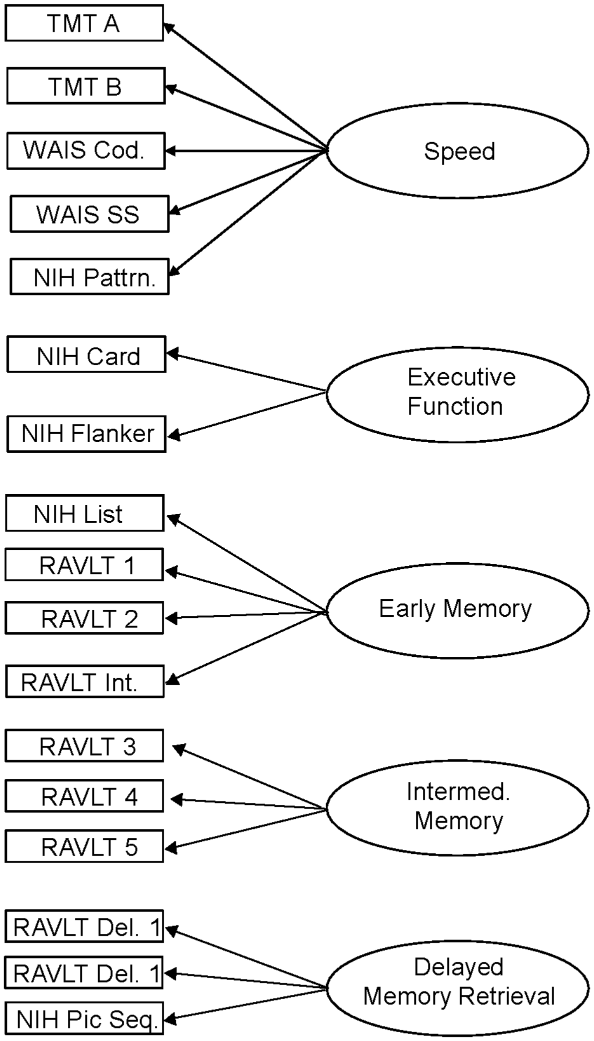

Factor model of cognitive performance measures assessed at 2 weeks post-injury. Five factors were modeled as correlated. The 15 indices derived from the model reflected those of the following National Institutes of Health TBI Common Data Elements: Rey Auditory Verbal Learning Test (RAVLT, comprising 5 learning immediate recall trials and 1 interference immediate recall, 1 short-delay recall, and 1 long-delay recall trial); NIH Toolbox Picture Sequence Memory subtest;[2] Wechsler Adult Intelligence Scale–Fourth Edition (WAIS-IV) Coding and Symbol Search subtests;[3] NIH Toolbox Pattern Comparison Processing Speed, Trail Making Test Parts A and B; NIH Toolbox Dimensional Change Card Sort, Flanker Inhibitory Control, and Attention subtests.[4,5]

© 2021 Brett BL et al. *JAMA Network Open.*

eAppendix 1. Factor Analysis of Cognitive Measures

It was expected that the multiple cognitive measures assess overlapping constructs, and as such, a data reduction strategy (i.e., factor analysis) was employed. To similarly reduce the 15 indices of the cognitive performance battery into subdimensions, cognitive measures were submitted to exploratory (EFA) in a random split half sample using maximum likelihood estimation with robust standard errors estimation. This estimation approach accommodates item-level missingness with full information maximum likelihood, which has been demonstrated to be robust to missing at random missingness mechanisms. Due to the fact that cognitive abilities are often strongly intercorrelated, EFA analyses emphasized correlated-factor models (using GEOMIN rotation). A set of CFA models were developed from the EFA model results in the second split half random sample, placing each item on one factor based on a combination of its loading pattern, prior findings for a given instrument, and theoretical considerations. Model fit was considered excellent if root mean squared error of approximation (RMSEA) < .06 and comparative fit index (CFI) and Tucker Lewis index (TLI) were > .95 and was considered acceptable if RMSEA < .08 and CFI/TLI > .90.[6] Factors scores for the final factor model were extracted for the full sample for use in LPA analysis.

eAppendix 2. Criteria for Determining Final Latent Profile Analysis Model

Determining the optimal profile model (i.e., number of unique profiles) was based on relative fit statistics. Simultaneous consideration of the Bayesian information criterion (BIC)[7] index and modified Lo-Mendell-Rubin (LMR) likelihood ratio tests (LRTs).[8] A non-significant outcome for the LMR LRT suggests that a solution with *k* latent classes should be selected, as extraction of an additional class (*k*+1) did not significantly improve model fit. Given that BIC tends to improve with increasing number of extracted classes, optimal class solution (i.e., number of profiles) was also determined based on additional considerations such as interpretability, non-trivial class sizes, and identification of qualitatively distinct classes.

eAppendix 3. Range and Interpretation of Distal Outcomes at 6 Months After TBI

Satisfaction With Life Scale (SWLS; possible range, 0–35; higher scores indicate more life satisfaction);[9] Quality of Life after Brain Injury-Overall Scale (QoLIBRI-OS; possible range 0–100; higher scores indicate more life satisfaction);[10] Rivermead Post Concussion Symptoms Questionnaire (RPQ) Total score (range 0–64; higher scores reflect more new/worsened symptoms since injury); Glasgow Outcome Scale-Extended (GOSE; range 1–8, with 8 indicating better injury recovery).[11] GOSE scores reflected reports of any changes in functioning since injury, whether it was perceived to be due to TBI or peripheral injuries.

eAppendix 4. Propensity Weighting Procedure and Related Variables

Propensity weighting was utilized in order to account for potential bias due to missingness of 6-month outcomes. For each of the 6-month outcomes (SWLS, QoLIBRI-OS, RPQ, and GOSE), individual propensity models were constructed for the

© 2021 Brett BL et al. *JAMA Network Open.*

presence/absence of the each outcome based on the following variables: age, education, emergency department Glasgow Coma Scale score, Alcohol Use Disorders Identification Test-Concise (AUDIT-C) score, enrollment site, level of care, sex, race, ethnicity, pre-injury living situation, insurance type, employment status, primary first language, injury cause, major extracranial injury, previous TBI, loss of consciousness, post-traumatic amnesia, psychiatric history, CT positive finding, tobacco smoker, drug use history. Using the TWANG boosted regression algorithm (available at the rand.org website) and the above variables, the resulting propensities for the presence of the outcome were determined. Weights for the analyses were calculated by reciprocating each propensity score and standardizing such that the average weight corresponding to each type of outcome measure remained equal to one in order to preserve the original sample sizes.

eAppendix 5. Factor Analysis Results of Cognitive Measures

In Half 1 of the data, EFAs were performed, extracting 1 to 12 factors with an oblique (geomin) rotation. A 5-factor solution (**Supplemental eFigure 3**) was deemed to have the best balance of increased fit (compared to extracting fewer factors) and parsimony (compared to extracting more factors). From largest eigenvalue to smallest, the identified factors were labeled as: 1) Speed, 2) Executive Function, 3) Early Memory, 4) Intermediate Memory 5) Delayed Memory. A correlated 5-factor CFA was developed based on EFA results and run on Half 2 of the data, which demonstrated good model fit, $\chi^2(109) = 866.25$, $p < .001$, CFI = .95, TLI = .94, RMSEA = .067 (90% CI = .063, .071).

eAppendix 6. Results of the Latent Profile Analyses Model Fit

Model fit significantly improved when increasing the number of profiles from 2 to 3 to 4, LMR LRT *ps* < .0001. Modeling 5 groups did not significantly improve fit (p=.185). As expected, BIC values decreased (i.e., reflected improved fit) with an increasing number of LPs. Visual inspection of BIC values showed a minimal fit improvement beyond 4 profiles, supporting LRT results. Within the 4-profile solution, the average probabilities for the most likely LP membership, or the quality of class allocation, were 0.94, 0.90, 0.93, and 0.96. Relative entropy indicated good delineation of classes (.89).

© 2021 Brett BL et al. *JAMA Network Open.*

eTable. Overall and Relative Fit of Models With Increasing Number of Latent Profiles

Latent Profiles Number	Free Parameter	BIC	AIC	H0 Log-likelihood	log-likelihood difference*	LMR LRT p-value#	Entropy
1	22	51808	51688	-25822.018			
2	34	46836	46650	-25822.018	5061.676	<.001	.866
3	46	44315	44063	-23291.179	2610.635	<.001	.895
4	58	42024	41707	-21985.862	2354.293	.0002	.885
5	70	41004	40621	-20795.586	1097.503	.1845	.883
6	82	40284	39835	-20240.714	800.755	.1894	.875

AIC=Akaike's information criterion; BIC=Bayesian information criterion; LMR LRT =Lo–Mendell–Rubin likelihood ratio test; *Difference between models of (k – 1) and k classes; # A p-value <0.05 suggests that a model with k classes fits significantly better than a model with k-1 classes, according to the Lo-Mendell-Rubin (LMR) Adjusted LRT. The model with 4 classes was selected based on the LMR-LRT results, interpretability, non-trivial class sizes, and identification of qualitatively distinct classes. Criteria for deciding on the number of latent classes included BIC and LMR LRT based on a Monte Carlo simulation study. The final 4-profile model selected highlighted in bold.

© 2021 Brett BL et al. *JAMA Network Open.*

eReferences

1. Nelson LD, Kramer MD, Joyner KJ, et al. Relationship between transdiagnostic dimensions of psychopathology and traumatic brain injury. *Under Review.* 2020.
2. Schmidt M. *Rey Auditory Verbal Learning Test: A handbook.* Loss Angeles, CA: Western Psychological Services; 1996.
3. Wechsler D. *Wechsler Adult Intelligence Scale.* 4th ed. San Antonio, TX: Psychological Corporation; 2008.
4. Reitan RM. Trail making test results for normal and brain-damaged children. *Percept Mot Skills.* 1971;33(2):575-581.
5. Reitan RM, Wolfson D. *The Halstead-Reitan Neuropsychological Test Battery: Therapy and clinical interpretation.* Tucson, AZ: Neuropsychological Press; 1985.
6. Hu L-t, Bentler PM. Cutoff criteria for fit indexes in covariance structure analysis: Conventional criteria versus new alternatives. *Structural Equation Modeling.* 1999;6:1-55.
7. Schwartz G. Estimating the dimension of a model. *The Annals of Statistics.* 1978;6:461-464.
8. Lo Y, Mendell N, Rubin D. Testing the number of components in a normal mixture. *Biometrika.* 2001;88:767-778.
9. Diener E, Emmons RA, Larsen RJ, Griffin S. The Satisfaction With Life Scale. *J Pers Assess.* 1985;49(1):71-75.
10. von Steinbuchel N, Wilson L, Gibbons H, et al. Quality of Life after Brain Injury (QOLIBRI): scale development and metric properties. *J Neurotrauma.* 2010;27(7):1167-1185.
11. Wilson JT, Pettigrew LE, Teasdale GM. Structured interviews for the Glasgow Outcome Scale and the extended Glasgow Outcome Scale: guidelines for their use. *J Neurotrauma.* 1998;15(8):573-585.

© 2021 Brett BL et al. *JAMA Network Open.*
